# Highly sensitive fiber-optic accelerometer by grating inscription in specific core dip fiber

**DOI:** 10.1038/s41598-017-12322-6

**Published:** 2017-09-19

**Authors:** Qiangzhou Rong, Tuan Guo, Weijia Bao, Zhihua Shao, Gang-Ding Peng, Xueguang Qiao

**Affiliations:** 1 Department of Physics, Northwest University, Xi’an, 710069 China; 2 Guangdong Provincial Key Laboratory of Optical Fiber Sensing and Communications, Institute of Photonics Technology, Jinan University, Guangzhou, 510632 China; 3 School of Electrical Engineering and Telecommunications, University of New South Wales, Sydney, NSW 2052 Australia

## Abstract

A highly sensitive fiber-optic accelerometer based on detecting the power output of resonances from the core dip is demonstrated. The sensing probe comprises a compact structure, hereby a short section of specific core (with a significant core dip) fiber stub containing a straight fiber Bragg grating is spliced to another single-mode fiber via a core self-alignment process. The femtosecond laser side-illumination technique was utilized to ensure that the grating inscription region is precisely positioned and compact in size. Two well-defined core resonances were achieved in reflection: one originates from the core dip and the other originates from fiber core. The key point is that only one of these two reflective resonances exhibits a high sensitivity to fiber bend (and vibration), whereas the other is immune to it. For low frequency (<10 Hz) and weak vibration excitation (<0.3 m/s^2^) measurement, the proposed sensor shows a much higher resolution (1.7 × 10^−3^ m/s^2^) by simply monitoring the total power output of the high-order core mode reflection. Moreover, the sensor simultaneously provides an inherent power reference to eliminate unwanted power fluctuations from the light source and transmission lines, thus providing a means of evaluating weak seismic wave at low frequency.

## Introduction

Monitoring weak vibration (acceleration) at low frequency is a critical issue in various engineering applications, especially for seismic exploration and oil well production^1,2^. For more than two decades, fiber-optic accelerometers have attracted widespread interests because of their unique properties^3,4^; fiber-optic accelerometers have enabled a multitude of opportunities for single-point vibration sensing in hard-to-reach spaces, with controllable cross-sensitivities and very compact size for embedded measurement. The traditional interrogation method for fiber Bragg grating (FBG) sensors, which is based on wavelength monitoring, suffers from high cost and low interrogation speed^5–7^. To simplify the interrogation scheme and achieve a real time output, methods using power detection have been studied thoroughly, along with a variety of FBG sensor configurations. Because cladding modes have unique mode field shapes, they show different responses to perturbations inside and outside the fiber (especially to transversal strain)^8,9^, providing good potential for enabling highly sensitive vibration measurement. However, when utilizing cladding modes, a suitable coupling mechanism must be provided to recouple the cladding mode back into the upstream core to allow remote interrogation with low loss. In prior reports of fiber grating vibroscopes, the specific post-processes (offset splicing^10^, abrupt taper^11,12^, and core mismatching^13–15^) have been proposed to address this function. To avoid the issues of weakened mechanical strength (abrupt taper), low reproducibility (offset splicing) and complex fabrication process (specific post-processes), our group has proposed a method named “cladding FBG” that involves the simple writing of a conventional FBG in the cladding of a short piece of thin-core fiber (TCF)^16,17^. With this configuration, an expected single cladding mode resonance appears in the reflection spectrum that can be utilized in directional acceleration measurements. However, the downsides of such a configuration are the enhanced insert loss and the relatively low vibration sensitivity.

**Figure 1:**
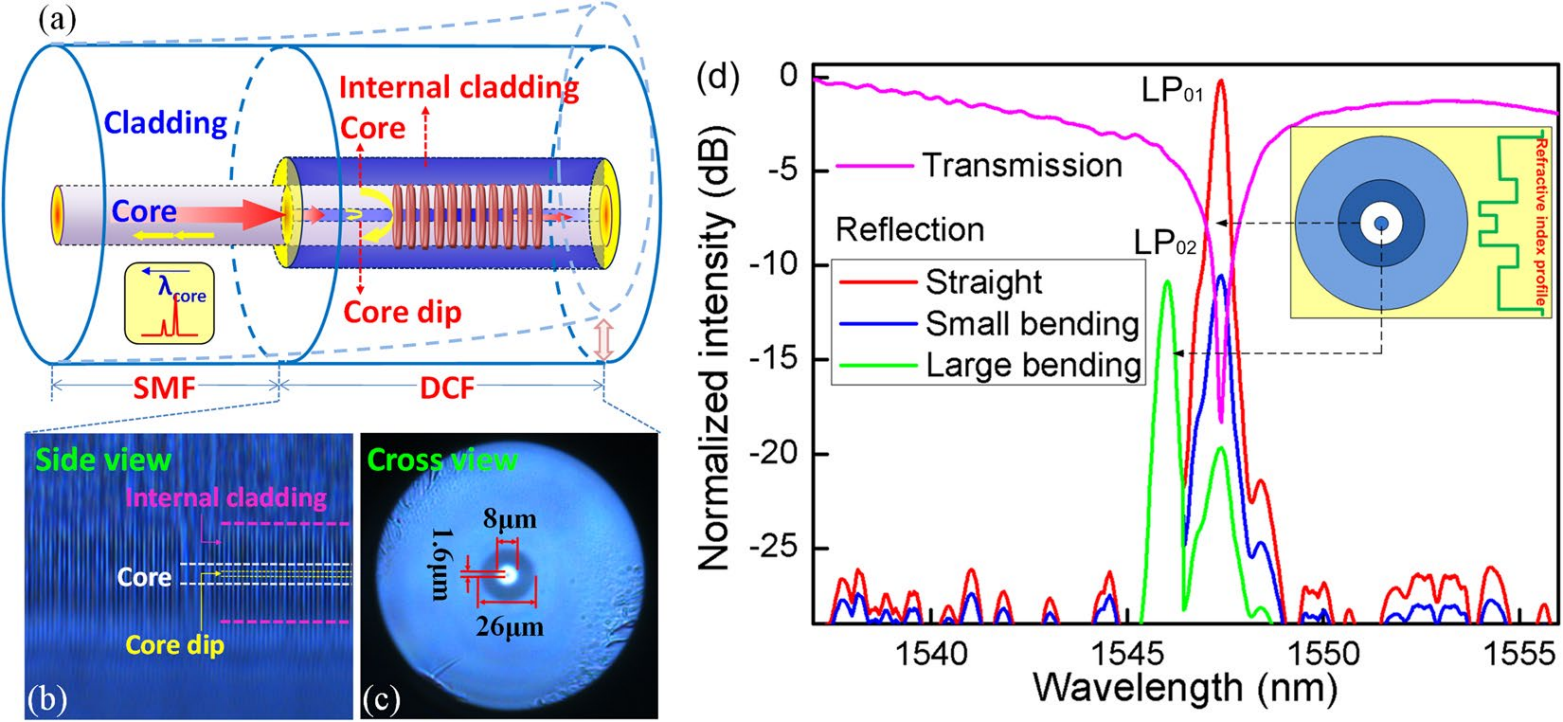
(**a**) Schematic of the DCF-FBG accelerometer, (**b**) side-view images of the DCF with grating inscription, (**c**) cross-view image of DCF, (**d**) transmission and reflection spectra of DCF-FBG versus fiber bending, where on the short wavelength side the green line overwrites the previous ones (red and blue lines). Inset shows the transverse refractive index distribution.

In this paper, we propose an improved vibration sensing mechanism that retains a simple configuration, i.e., a specific core (with a core dip) fiber^18,19^ is employed for FBG inscription, as shown in Fig. 1. The key aspect of this device is the inherent index profile of the fiber (which restrains the generation of core modes) and the precisely localized grating inscription over the fiber core (via high-intensity focused femtosecond laser pulses and the photosensitivity of depressed-cladding fiber (DCF) core). As described below, such a device (a compact sensor probe with a well-defined high-order core mode reflection) provides high sensitivity for monitoring weak vibration (acceleration) at low frequency, together with a tunable resonance frequency and simplified power-referenced detection.

## Results

Figure 1 shows a schematic of the DCF-FBG, side-view image of the DCF with grating inscription and spectral performance. The photo of the DCF cross section by optical microscope is shown in Fig. 1(c). The sensor is constructed by a SMF spliced with a section of photosensitive DCF of several centimeter long. The DCF has a core diameter of 8 μm, with a dip inside the core of 1.6 μm diameter, and an outer cladding diameter of 101 μm, with a depressed inner cladding of 26 μm diameter. As shown in Fig. 1(a), the grating is inscribed in the DCF section where the fiber core with a significant dip.

The grating inscription is performed using well-established phase-mask technology. The details of the fabrication process are provided in the following Methods section. The grating length is approximately 2 mm, and the grating pitch is approximately 0.5 µm. According to the magnified photographic images shown in Fig. 1(b), the fiber exhibits periodic “line” like pattern (i. e. grating pitches). Once the grating is achieved, two well-defined resonances in reflection are observed because of the difference between the effective indices of core and the core dip of the DCF (approximately 0.00278), as shown in Fig. 1(d). The reflection spectrum is measured with a rough sensing end-face to avoid unwanted reflection from the fiber end-face. Among these two core resonances in reflection, the fundamental core resonance (LP_01_ mode) are located at the longer wavelength side with a 1.4 nm band gap away from the high-order core resonance (LP_02_ mode, caused by the refractive index difference between the core and the core dip, as the inset shown in Fig. 1(d)). The coupling between LP_01_ mode and LP_02_ mode (that can be ensured by resonance wavelengths separation in spectrum and refractive indices of fiber cross-section) strongly occurs experimentally for this particular grating. Compared with earlier reports of ghost modes in a tilted FBG (a group of low-order cladding modes overlapping each over^10^), the DCF-FBG spectrum, showing a clean spectrum with a sole mode and much improved signal-to-noise ratio, has good potential for orientation-independent measurement and sensor multiplexing.

The excellent spectral performance is attributed to the refractive index profile of the fiber cross-section. The depressed cladding (with a suitable width and refractive index), together with changing the boundary conditions and forming a weak “pinning” effect, helps reduce the cladding mode field overlapping in the core region, and ultimately reduces the coupling and recoupling of the core-to-cladding modes in DCF^20^. Moreover, because the transverse electric field amplitude distribution of this high-order core mode is strictly confined to outer fiber core via the depressed cladding (mainly focused in 8.0 μm in diameter) and can be well recoupled into the upstream SMF fiber (with a core 8 μm in diameter), it is insensitive to small degree of fiber bend. By contrast, the fundamental core mode at the longer side (with a higher initial power intensity) exhibits a very high sensitivity to fiber bend because of its larger transverse electric field amplitude distribution (can be greater than 1.6 μm in diameter), which can easily “leak” out, resulting in a very lossy core to enable core coupling to the upstream SMF. This recoupling loss of fundamental core has been identified by the result shown in Fig. 1(d), whereby the spectral intensity of the fundamental core mode decreases sharply with fiber bend, whereas the high-order core mode remains unchanged. As a result, a highly sensitive vibration (acceleration) measurement can be achieved via cost-effective power detection of the fundamental core mode resonance. Moreover, the unwanted power fluctuations and temperature perturbations can be referenced out by monitoring the high-order core mode resonance.

**Figure 2:**
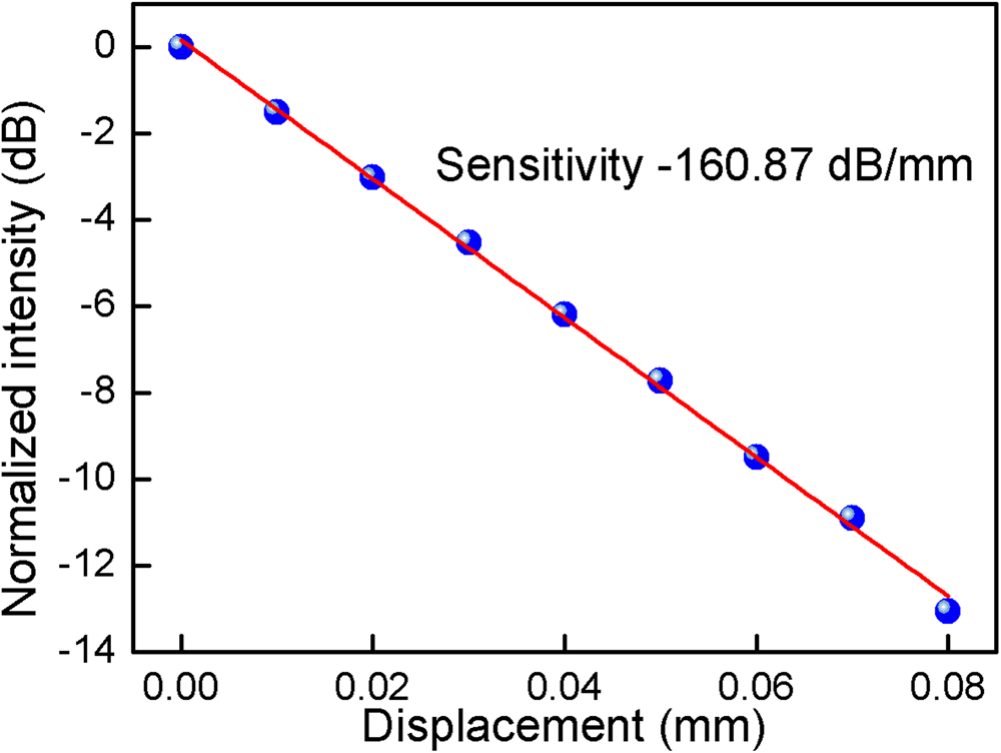
Linear response of the sensor output versus the applied displacement.

**Figure 3:**
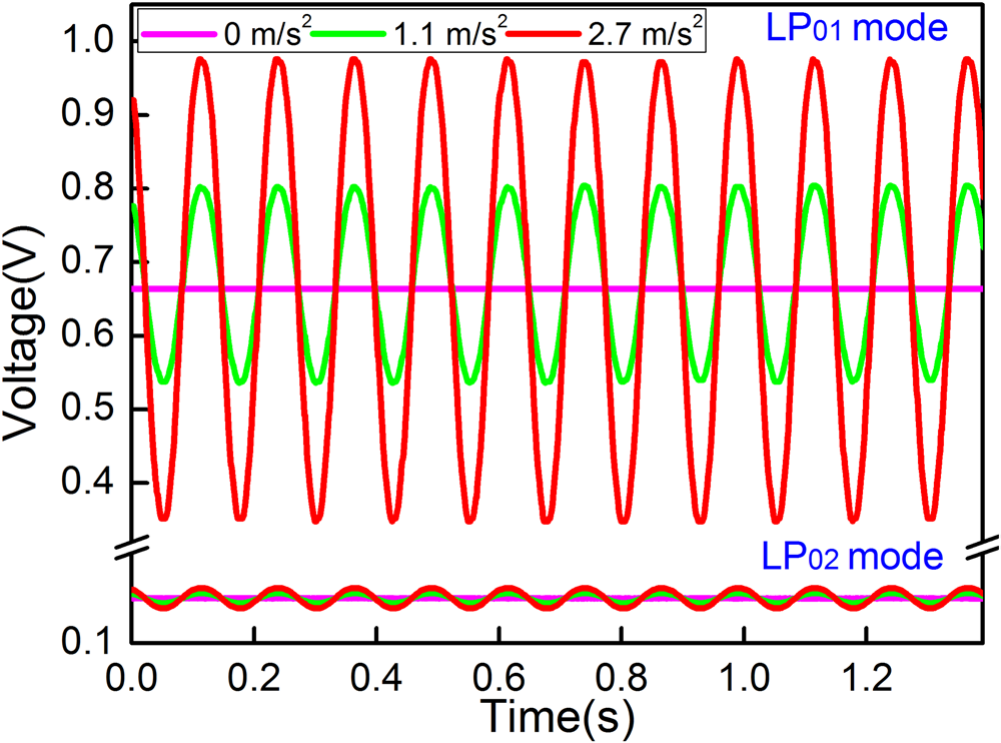
Real-time power output of the two reflective core resonances versus applied vibration with three acceleration values.

Bending or deflecting the fiber introduces refractive index variations across the fiber that influence the reflection spectrum in the following ways: the forward fundamental mode coupling to the core at the SMF-DCF splicing junction (forward coupling loss); the propagation loss of the core modes between the splicing junction and the downstream FBG (bend loss) caused by the weak restraining power ability of the DCF depressed cladding; and the backward fundamental core mode field recoupling at the SMF-DCF splicing junction (mostly caused by the stress induced refractive index variation changes along the fiber cross section that would compensate or increase the mode offsets at the junction). As a result, bending the fiber will induce a strong intensity modulation over the recoupled fundamental mode. To further demonstrate the bend sensitivity of the fundamental mode resonance, the upstream side of the sensing fiber is held on a fixed stage, and then the FBG is bent by elevating the free fiber end using another lifting stage (using the same operation process described in our prior work^14^). Figure 2 shows the peak intensity loss of the fundamental mode resonance as a function of the displacement (bend), revealing that a high sensitivity of −160.87 dB/mm is achieved. This sensitivity is two orders of magnitude higher than that of the low-order odd cladding mode reported in the previous work^14^. Another result is real-time power output of the core mode shown in Fig. 3 (under a 16 Hz harmonic oscillation), which highlights the performance of dynamic vibration measurement. The effect mentioned above explains the change in reflected high order core mode, especially in view of the fact that the recoupled power decreases and increases around its unbent stage, as indicated by the oscillating curve shown in Fig. 3.

**Figure 4:**
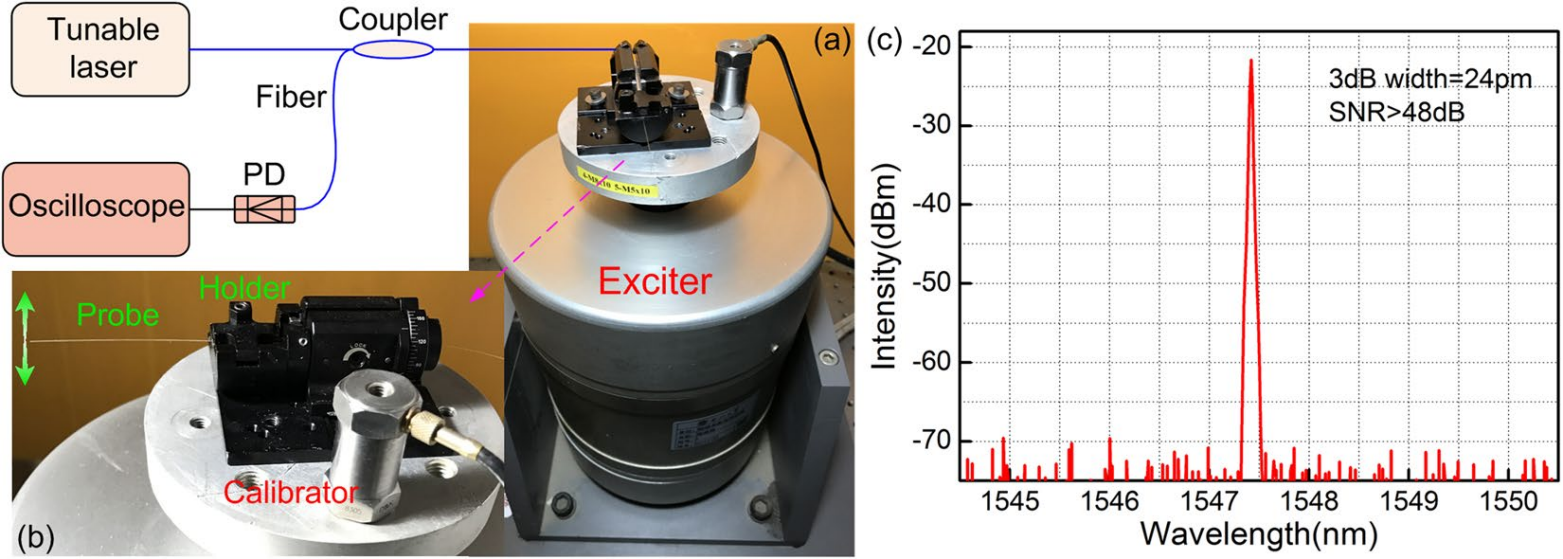
(**a**) Schematic of the acceleration sensing system, (**b**) magnified view of the DCF-FBG sensing probe and the acceleration calibrator, and (**c**) reflection spectrum as laser is fixed at the fundamental mode position.

The schematic configuration of the acceleration sensing system is shown in Fig. 4(a). A tunable laser (Santec 710) with 100 kHz linewidth and 0.1 pm tunable resolution is employed as the light source; the laser is launched into the sensing probe through a circulator. The laser wavelength is fixed at the peak position (1547.4 nm) of the high-order core mode resonance, and the laser output has a narrow spectral bandwidth (24 pm) and a high signal-to-noise ratio (>48 dB). The real-time power of reflected laser beam is modulated by the high-order core mode resonance loss resulting from the dynamical fiber bend. The real-time power output is monitored by a photodiode (PD, New Focus) with a bandwidth of 10 MHz at 0 dB gain coupled to an oscilloscope for signal analysis. The sensing probe is held at an exciter with a fiber holder to provide different accelerations. The free fiber length downstream must be carefully selected because it functions as an inertial mass, which dominates the sensor resonance frequency and amplitude-frequency response.

**Figure 5:**
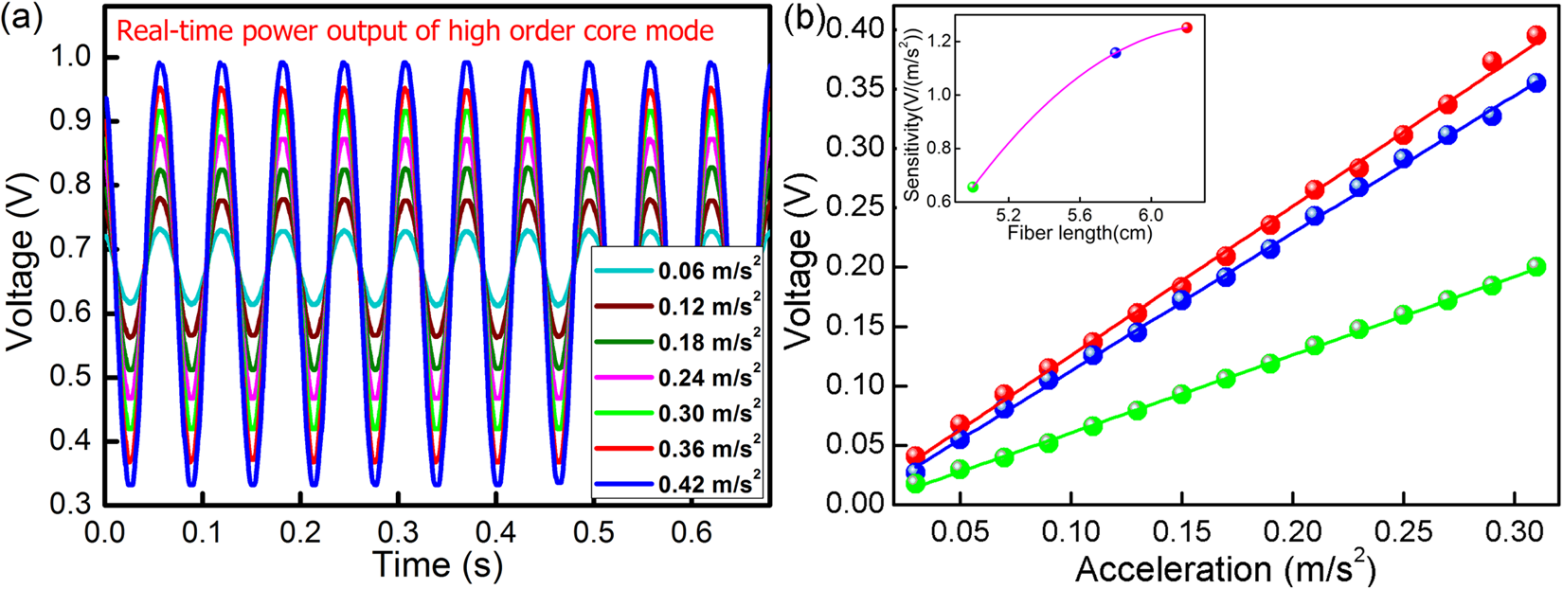
(**a**) Real-time power output of the fundamental core mode versus the applied acceleration, (**b**) linear response output of sensors with different free fiber lengths versus the applied accelerations, whereby inset shows the sensitivity versus the sensor length.

A series of sine vibration waves with acceleration amplitudes ranging from 0.03 m/s^2^ to 0.31 m/s^2^ and an interval step of 0.02 m/s^2^ is applied to 6.2 cm-long sensor, for the case of the vibration frequency maintained at 16 Hz. The vibration signal output is calibrated using a standard piezoelectric accelerometer with a sensitivity of 1.26 pC/G and frequency band of 1 Hz to 5000 Hz produced by the company of Beijing Wavespectrum Science & Technology, China. The sine curves in Fig. 5(a) present the reflected power of the core mode versus different accelerations in the time domain. The patterns of the curves are similar to that in Fig. 3, which importantly identifies the sensor’s ability to operate well in the high frequency region well. Figure 5(b) shows that the peak amplitude of the high-order core mode is proportional to the acceleration, with a highly linear sensitivity of 1.15 V/(m/s^2^) (as shown by the blue line), which is one order of magnitude higher than that of cladding mode-based accelerometer described in our prior work^16^. According to the power detection resolution (2 mV) of the PD, the demonstrated resolution of the acceleration measurement is calculated as 1.7 × 10^−3^ m/s^2^. To know on how the sensing length (the total length from the fixed point to the fiber tip) would influence sensor performance, the response to acceleration for three different sensing lengths (L = 6.2 cm, 5.8 cm and 5.0 cm) is characterized. The experimental results are shown in Fig. 5(b), while the inset in Fig. 5(b) presents the response versus the sensing length. The inset in Fig. 5(b) presents the sensor sensitivity versus the sensor length. Furthermore, the amplitude-frequency response curve is one of the key characteristics of the accelerometer as it demonstrates the effective measurable frequency range (flat bandwidth) and the quality (flat equalization) of the proposed sensor.

**Figure 6:**
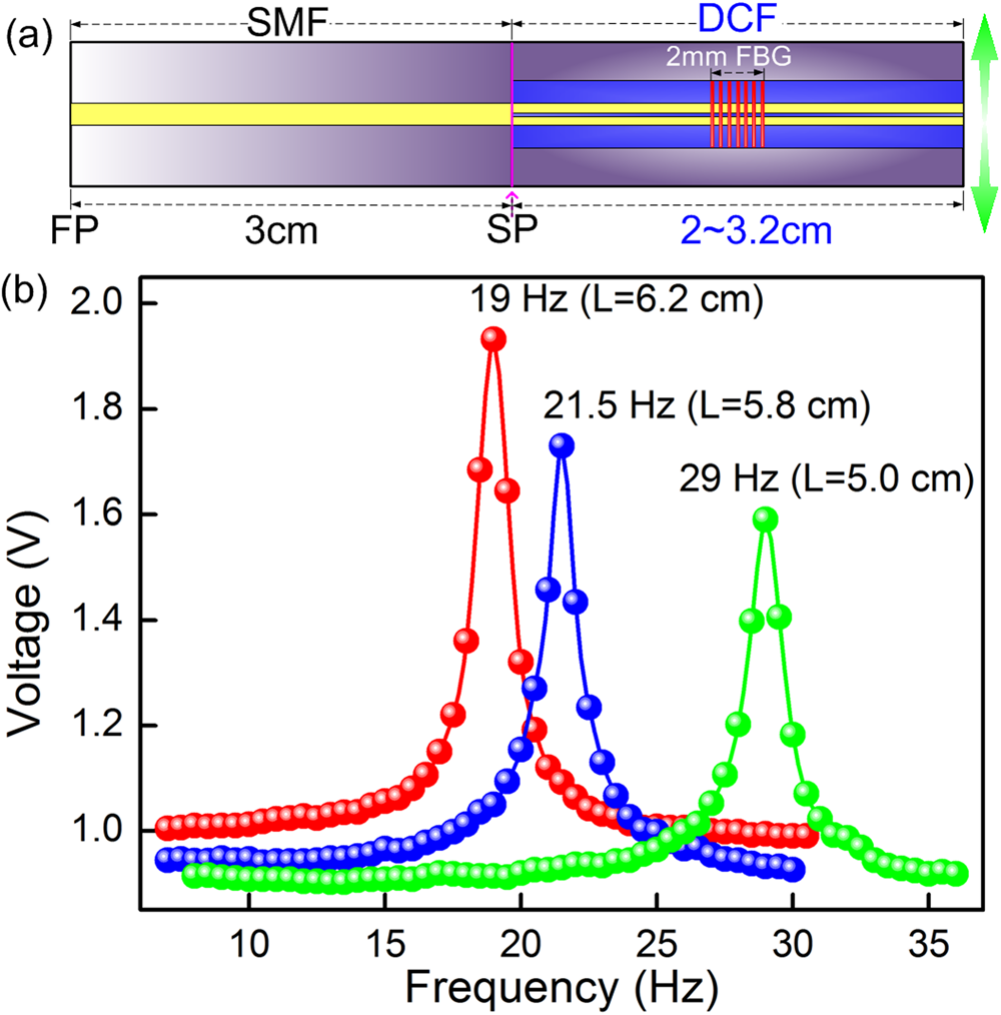
(**a**) The schematic sensor configuration, FP is the fixed point on fiber holder and SP is the splicing point; (**b**) The amplitude-frequency response of the sensors with different free lengths.

The configuration of the sensor head is depicted in Fig. 6(a). The sensing length consists of a section of SMF starting from the fixed point (FP) to the splicing point (SP) and a section of DCF from the SP to the fiber tip. The DCF section contains a 2 mm FBG. Of the three experimental sensors tested, the SMF section has the same 3 cm length, while the DCF section has different lengths between 2 and 3.2 cm, i.e. 2.0 cm, 2.8 cm and 3.2 cm, respectively. Hence the total sensing length is correspondingly 5 cm, 5.8 cm and 6.2 cm. Figure 6(b) presents the reflected amplitude versus the oscillation frequency at a fixed acceleration of 0.07 m/s^2^. As shown by the blue points in Fig. 6, a resonant frequency peak of 21.5 Hz for the 5.8 cm sensor appears in the test range of 7 Hz to 30 Hz, with the frequency increasing at an interval of 0. 5 Hz. The blue and green curves show the frequency responses of two other different length sensors, for which the resonant frequencies are effectively tuned to the lower- (higher-) frequencies with the increase (decrease) of the sensor length: 19 Hz for the 6.2 cm sensor and 29 Hz for the 5.0 cm sensor. However, compared with the results shown in Fig. 6(b), note that there is a trade-off between the resonant frequency and the response sensitivity.

## Discussion

### Sensing mechanism

Modeling of optical fibers typically relies on the translation invariance of the fiber’s refractive index distribution in the propagation direction, *z*, of light. When the fiber is bent, an additional perturbation of the refractive index profile occurs across the fiber cross section, leading to two effects^21,22^: the mode fields shift laterally along the bend plane direction and an additional term must be included in the refractive index perturbation used in the coupling integral. Different from the standard SMF, the transverse E-field amplitude distribution of this high-order core mode (LP_02_) is well confined in the fiber core because of the step-index distribution between core and depressed-cladding. As a result, this circularly symmetrical mode can be well recoupled into the upstream SMF fiber (with a core of 8 μm in diameter that is well core-matched with the upstream SMF), therefore it is insensitive to small degrees of fiber bend. The fundamental mode (LP_01_) at the longer side has the higher initial power intensity because of the smaller E-field distribution (caused by the 1.6 μm diameter fiber dip) is well recoupled to the upstream SMF. Moreover, because of the weak waveguiding condition between the core dip and the core, the power of the fundamental mode is easily disturbed by the bent fiber; therefore, in contrast to the high-order mode, the fundamental mode presents a much higher sensitivity to fiber bend. The E-field of fundamental mode of bent fiber is easy to “leak” out and therefore induces a large reflected power loss. In order to better understand, fiber bend modeling and simulation are characterized as follows.

**Figure 7:**
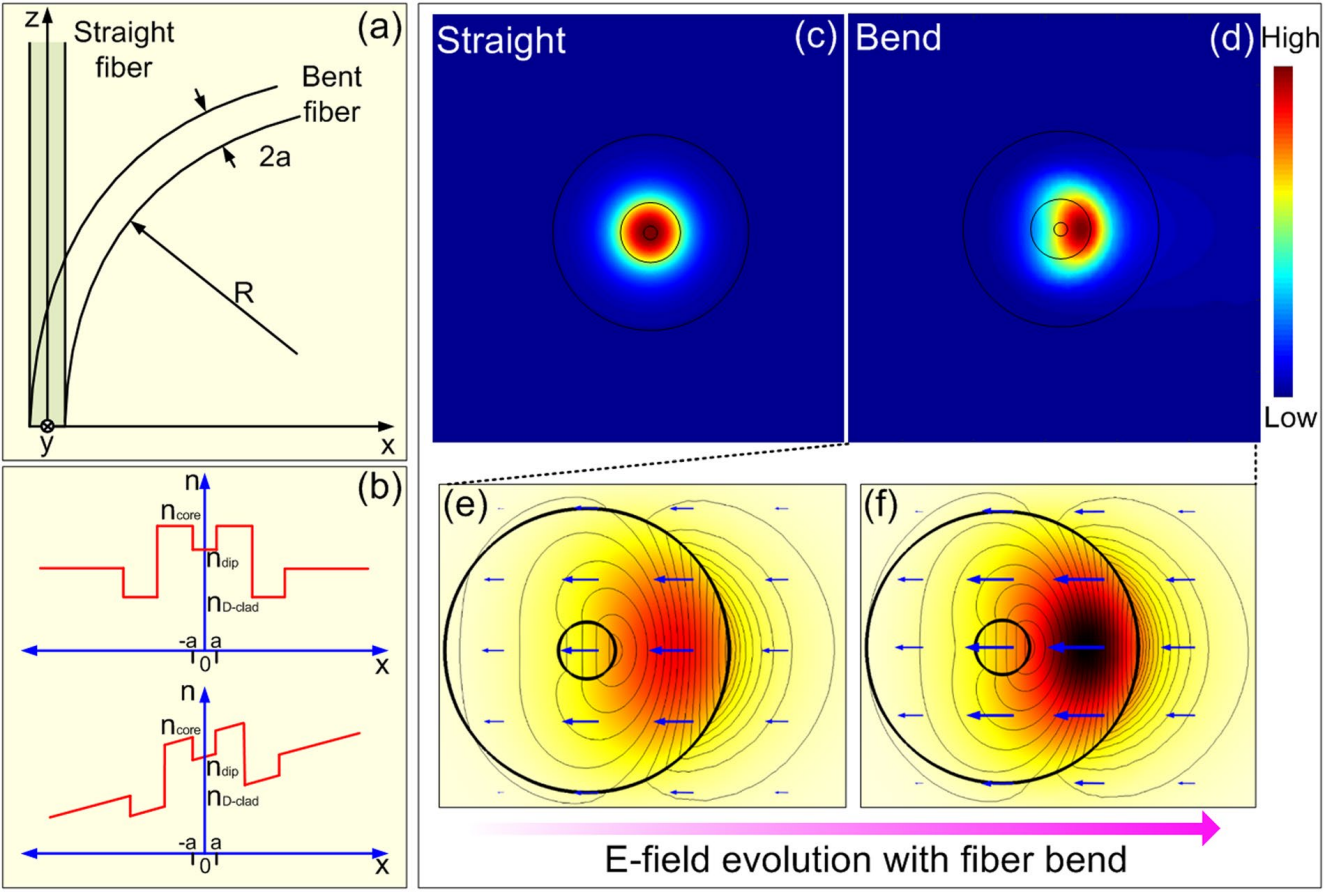
(**a**) Schematic of a circularly bent fiber, (**b**) Refractive index distribution of an unstressed (up), bent fiber (down), (**c**) E-field distribution of straight fiber, (**d**) E-field distribution of bent fiber in a certain degree, and (**e**) and (**f**) magnified schematic evolution of the fundamental mode with fiber bend (the average power of the E-field is forced to move out the fiber dip and eventually loss out fiber).

As shown in Fig. 7(a), if the cross-section of the fiber is set to x-y plane, coordinate transformation allows the bent fiber to be expressed, with modified refractive index distribution^23^

1 $$n\text{'}(x,y)={n}_{material}(x,y)\exp (\frac{x}{R})\approx {n}_{material}(1+\frac{x}{R})$$

Here $x\ll R$ is assumed with respect to relatively slow bends, ${n}_{material}(x,y)$ is the refractive index of bent waveguide cross-section, and $R$ is the bend radius. As the bend-induced stress-optic effects are considered, compression along the inner half of the fiber, towards the center of the bend, and tension along the outer half, cause the material refractive index to vary (shown in Fig. 7(b)) according to the relation

2 $$n\text{'}(x,y)=n(x,y)\{1-\frac{{n}^{2}x}{2R}[{P}_{12}-\nu ({P}_{11}+{P}_{12})]\}$$

Here $n(x,y)$ is the refractive index of the straight fiber, $\nu$ is Poissson’s ratio, and ${P}_{11}$ and ${P}_{12}$ are components of the photo-elastic (or elasto-optical) tensor. Accordingly combing equations (1) and (2), the equivalent bend radius as

3 $${R}_{eff}\equiv \frac{R}{1-\frac{{n}^{2}}{2}[{P}_{12}-\nu ({P}_{11}+{P}_{12})]}$$

Combing equations (1) and (3), we can get the final transformation of refractive index of fiber bend written as

4 $$n\text{'}(x,y)={n}_{material}(1+\frac{x}{{R}_{{\rm{e}}ff}})$$

When the refractive index profile of the fiber cross section varies with fiber bend, according to the above analysis, mode field will present the mode fields shift laterally. The E-field distribution of fundamental mode before and after fiber bend in a certain degree is simulated by the finite element analyses methods in^24,25^. As the shown in Fig. 7(c) and (d), the center of the E-field significantly deviate the initial position and the energy is leaked out the fiber dip as well. To see clearly, magnified schematic evolution of the fundamental mode of bent fiber are simulated, as shown in Fig. 7(e) and (f) whereas the leaky power of fundamental mode increase gradually with the increasing fiber bend.

**Figure 8:**
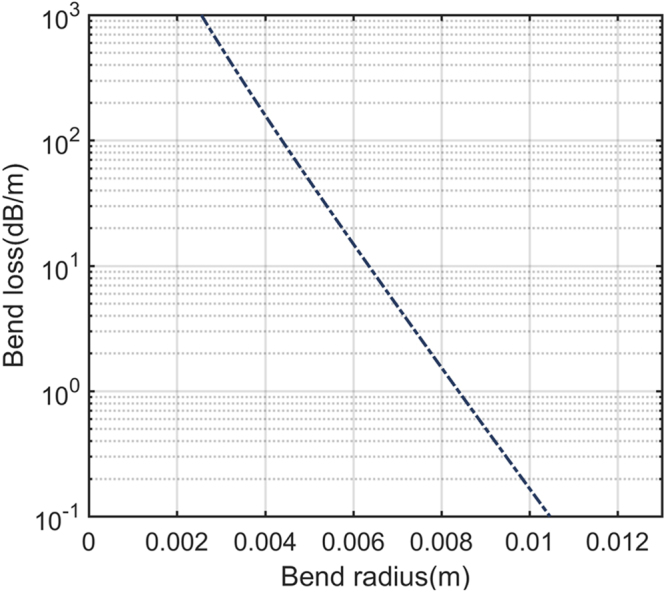
Analytically calculated loss using equation (4) of fundamental mode within the fiber core dip was analyzed.

As mentioned in^26–28^, regarding an analytical treatment of the bend losses in step-index fibers, only considering the fundamental mode’s response to fiber bend, a simplified bend loss formula can be expressed as

5 $$2\alpha [\frac{{\rm{dB}}}{{\rm{m}}}]=\frac{10/\mathrm{ln}(10){\pi }^{1/2}{\kappa }^{2}\exp [\frac{-2{\gamma }^{3}{(R+a)}_{eff}}{3{\beta }^{2}}-2\gamma a]}{{(R+a)}_{eff}^{1/2}{\gamma }^{3/2}{V}^{2}{K}_{m-1}(\gamma a){K}_{m+1}(\gamma a)}$$

where $a$ is the radius of the fiber dip core, $\beta$ is the propagation constant of the fundamental core mode, $\kappa =\sqrt{{n}_{core}^{2}{k}^{2}-{\beta }^{2}}$ and $\gamma =\sqrt{{\beta }^{2}-{n}_{clad}^{2}{k}^{2}}$ are the normalized propagation in the fiber dip and the cladding, respectively (where ${n}_{core}$ and ${n}_{clad}$ are the refractive indices of core and cladding, $k=2\pi /\lambda$ is the free space propagation constant), $V=\sqrt{{\kappa }^{2}+{\gamma }^{2}}$ is the normalized frequency, ${K}_{m\pm 1}$ is first modified Bessel function of the second kind. The fiber parameters used for the theoretical predictions are $2a$ = 1.6 µm, ${n}_{core}$ = 1.443 and ${n}_{clad}$ = 1.439 at the wavelength of 1550 nm, and $\beta$ and $V$ are achieved using the approach presented in^26,27^. Figure 8 shows the calculation result of bend loss versus bend radius using Eq. (5), where a linear function is achieved in units of (dB/m). As mentioned in^29^, the relation between acceleration applied is linearly proportional to the fiber bend in the vertical direction. The fundamental core mode should be linear function of applied acceleration, confirmed by the experimental demonstration shown in Fig. 5(b).

### Adjustable resonance frequency

**Figure 9:**
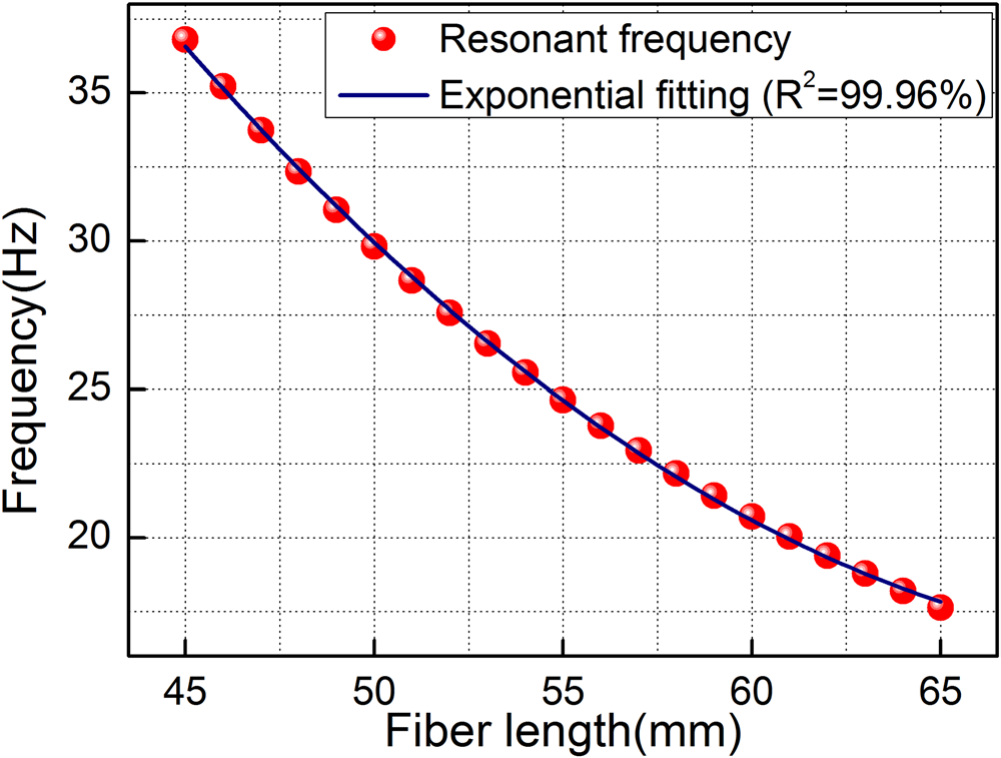
Theoretical resonance frequency versus free fiber length in theory.

The free fiber in the sensor can be regarded as a cantilevered beam. As mentioned in^30^, the resonance frequency of the sensor is given by

6 $${f}=\frac{1}{2\pi }\sqrt{\frac{8EI}{\rho A{l}^{4}}}$$

where $E$ = 73 GPa is Young’s modulus of fiber, ${l}$ is the length of the free fiber, $A=\pi {{r}}^{2}$ is the cross-sectional area of the fiber, ${r}$ = 125 µm is the radius of the fiber, $\rho$ = 2650 kg/m^3^ is the density of the fiber, and $I=\pi {{r}}^{4}/4$ is the moment of inertia of the fiber about the pivot. From equation (5), we can see that the resonant frequency can be increased by increasing the stiffness (effective Young’s modulus for the complete assembly), or by reducing the effective density, cross-sectional area or length of the sensor. Changing any of these parameters will result in a shift of resonant frequency of the sensor. However, the effect of ${l}$ is the parameter to which the resonant frequency is most sensitive and the easiest adjustable parameter for practical applications. Figure 9 shows the numerical calculation of resonant frequency versus free fiber length increasing from 45 mm to 65 mm. We can see that shortening the free fiber length is able to increase the resonant frequency of sensor; however shortening of the fiber length decreases the sensitivity of sensor, as indicated by the experimental results shown in Fig. 6. For a particular measurement, it is therefore important to select a suitable sensing fiber length by determining the frequency measurement range and vibrating sensitivity.

In conclusion, the proposed sensor comprises a grating inscription over the core and a core dip of a DCF that is fusion spliced to an additional piece of SMF. The entire sensor can be as small as 10 mm (possibly smaller for high-frequency vibration measurement) and does not require precise fabrication tolerances. The reflected power of the fundamental mode of the proposed sensor exhibits high sensitivity and high signal-to-noise ratio for fiber bends and vibrations in the low frequency range. Therefore, the interrogation scheme of the sensor is simplified by using the power detection (only requires one PD) instead of the traditional expensive wavelength monitoring instrumentation. Meanwhile, such power detection scheme also makes the sensor immune to temperature fluctuations of several tens of degrees Celsius as the reflected power of the fundamental mode is insensitive to the environmental temperature fluctuations^31^. Finally, the sensor is able to simultaneously provide an inherent power reference (by separately filtering the fundamental core resonance) to eliminate the unwanted power fluctuations (mainly originated from the light source and transmission lines).

## Methods

In detail, DCF-FBG inscription is performed by using a Ti: sapphire laser system, emitting linearly polarized light at 800 nm in the TEM00 spatial mode. The pulse length was 50 fs, with a beam diameter of <8.5 mm and a repetition rate of 1 kHz. The fs laser beam was precisely focused by an objective lens to a focal spot 5 μm in diameter. The average pulse energy of the laser output is fixed at 0. 5 mJ (controlled by an optical attenuator). The femtosecond laser beam is precisely focused along the core, then gratings in fiber core and core dip can be achieved simultaneously. Strong laser energy of Femtosecond laser (highly localized nonlinear light-material interaction) and photon-sensitivity of selected DCF contributes to this grating inscription. The exposure time lasts only 2 seconds (i.e. 2000 times laser pulses). Because of the fixed period of phase mask used in the experiment, the resonance spectrum of fundamental mode is posited at the wavelength close to 1550 nm. The DCF-FBG is spliced in a conventional fusion splicer after core alignment of two fibers. To avoid the fiber end-face reflection’s influence to resonance spectrum, the state of the sensing end-face is always keeping roughness. In order to interrogate the device, non-polarized light is injected to the grating by a tunable laser source with wavelength range from 1500 to 1600 nm, the spectrum of the sensor is detected by an optical spectrum analysis (OSA), and the dynamic power fluctuation of the sensor is detected by a power detector following with an oscilloscope.
